# Exergy Analysis of Directional Solvent Extraction Desalination Process

**DOI:** 10.3390/e21030321

**Published:** 2019-03-25

**Authors:** Sorour Alotaibi, Osama M. Ibrahim, Yu Wang, Tengfei Luo

**Affiliations:** 1 Mechanical Engineering Department, Faculty of Engineering and Petroleum, Kuwait University, Safat 13060, Kuwait; 2 Department of Aerospace and Mechanical Engineering, College of Engineering, University of Notre Dame, Notre Dame, IN 46556, USA; 3 Department of Chemical and Biochemical Engineering, College of Engineering, University of Notre Dame, Notre Dame, IN 46556, USA

**Keywords:** desalination, directional solvent extraction, octanoic acid, second-law analysis, exergy analysis

## Abstract

This paper presents an exergy analysis to evaluate the performance of a continuous directional solvent extraction (DSE) desalination process using octanoic acid. The flow of exergy was calculated for each thermodynamic state and balanced for different components of the system to quantify the inefficiencies in the process. A parametric study was performed to evaluate the impact of three critical design variables on exergy consumption. The parametric study reveals that the total exergy input decreases significantly with an increase in heat exchanger effectiveness. The results also indicate that the heat exchangers account for the highest exergy destruction. The total exergy consumption, however, has a slightly declining trend as the recovery-ratio increases. There is a small variation in the total exergy consumption, within the uncertainty of the calculation, as the highest process temperature increases. When compared to conventional desalination processes, the exergy consumption of the DSE, with heat recovery of 90%, is comparable to those of multi-stage flashing (MSF), but much higher than reverse osmosis (RO). Octanoic acid, which has low product water yield, is identified as the primary factor negatively impacting the exergy consumptions. To exploit the low-grade and low-temperature heat source feature of the DSE process, directional solvents with higher yield should be identified or designed to enable its full implementation.

## 1. Introduction

**Figure 1:**
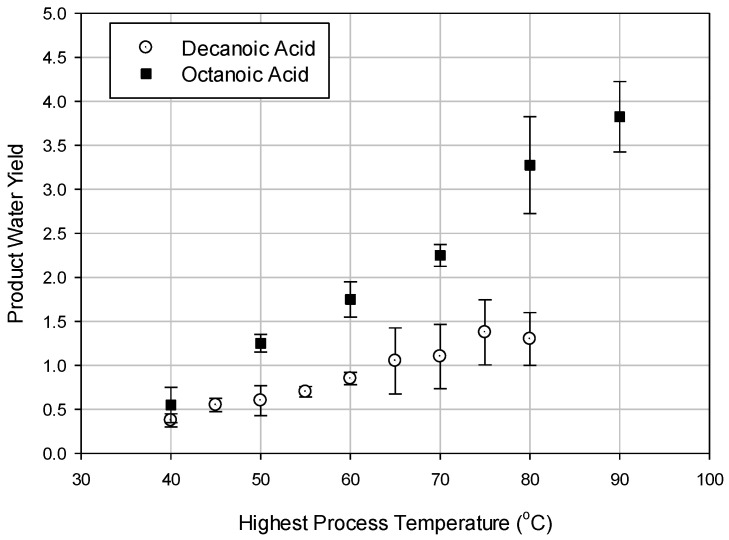
Product water yield of decanoic and octanoic acids vs. temperature [2].

Directional solvent extraction (DSE) is a newly proposed water desalination process that can separate pure water from seawater. This technique was first proposed by Davidson et al. [1] where pure water was separated using amines as directional solvents. Most preferred amine solvents were found to be the secondary or tertiary due to their high rejection of salt ions and their ability to absorb pure water, where the solubility of water increases with temperature. However, due to the high solubility of amine solvents in water the recovered water is often contaminated. Using this concept as the basis, Bajpayee [2] investigated the technical feasibility of this new technique using several directional solvents including octanoic and decanoic acids. The product water yield, which is the mass flow rate of purified water divided by the mass flow rate of the solvent, was measured at different temperatures for the most promising solvents: decanoic and octanoic acids, Figure 1 [2].

The properties of decanoic acid were studied by Bajpayee et al. [3], Luo et al. [4], Rish et al. [5], and Sanap et al. [6]. According to Rish et al. [5], using decanoic acid as a directional solvent, the ion rejection rates of DSE are in the range of 98–99%, which is close to the best reverse osmosis membranes used. In the work of Luo et al. [4], free energy calculations were used to study the solubility of water and decanoic acid, and molecular dynamics simulations were carried out to study their inter-diffusion processes. Their results confirmed that decanoic acid could absorb water and reject salt ions with very low solubility in water. Luo et al. [7] recently developed a continuous bench top DSE system realized by incorporating an electrocoalescer to speed up the water-solvent phase separation process. 

Assuming 80% heat recovery, the thermal energy consumption of a DSE process was estimated by Bajpayee [2] to be between 350–480 kWh/m^3^ for decanoic acid, and between 220–260 kWh/m^3^ for octanoic acid. Bajpayee, however, did not consider any electrical consumption associated with this new process. Sanap et al. [6] conducted an energy analysis of a continuous DSE process using octanoic acid. They predicted electrical energy consumption of more than 30 kWh/m^3^. This high electrical consumption was explained in their study by the high pumping power required to re-circulate the high mass flow rate of octanoic acid in a continuous desalination process. Alotaibi et al. [8] performed energy analysis for a DSE continuous water desalination process, with and without heat recovery. Assuming heat exchanger effectiveness of 0.8, the thermal energy consumption was estimated to be between 160 to 180 kWh/m^3^ for octanoic acid and between 350 to 460 kWh/m^3^ for decanoic acid; while the total electrical energy consumption is found to be between 5 to 9 kWh/m^3^ for octanoic acid and between 13 to 21 kWh/m^3^ for decanoic acid. This total electrical consumption includes the consumption of the pumps, mixing and that of the separation processes. The electrical energy required for the mixing process was predicted based on the power needed for a typical mixing impeller driven by an electrical motor. The additional electrical energy required to accelerate the separation processes, on the other hand, was estimated based on an energy efficient electrical demulsification. Their results for different high process temperatures, which were used as inputs to the process modeling, are summarized in Table 1.

In addition to the energy analysis, the exergy analysis is used to identify and quantify inefficiencies and to calculate the total exergy consumption of energy systems. The exergy analysis is considered an essential diagnostic tool in quantifying inefficiencies in energy processes, which can provide useful guidance on further system improvement. Several published papers considered exergy analyses of conventional desalination processes such as reverse osmosis (RO), multi-stage flash (MSF), and multi-effect distillation (MED), e.g., [9,10,11,12,13,14,15,16,17,18,19]. However, there are limited published technical papers on exergy analysis of the new DSE desalination process. Bajpayee [2] has performed a simplified exergy analysis on a DSE process by considering the temperature of the heat sources, where the analysis was first done by calculating the energy consumption of the batch process to estimate the energy and exergy requirements of the continuous desalination process. The results of the simple exergy analysis were compared to published results for RO and MSF. 

**Figure 2:**
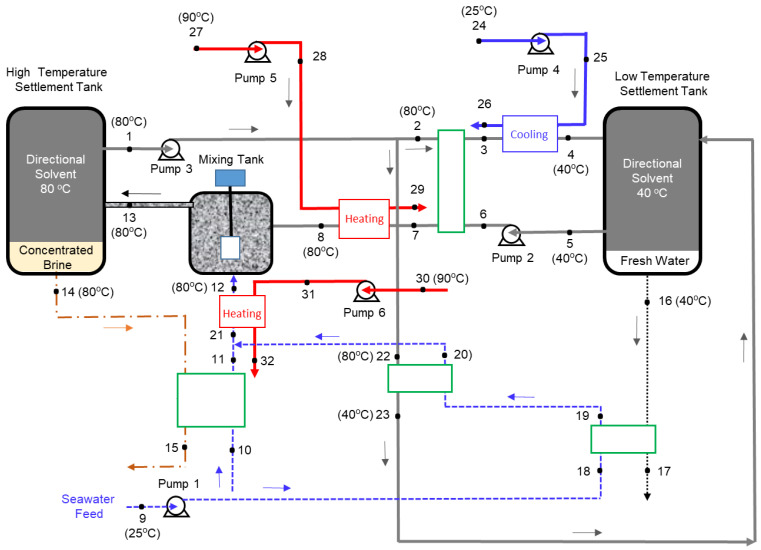
Optimized directional solvent extraction (DSE) process flow diagram [8].

The objective of this paper is to perform a detailed exergy analysis on a DSE process using octanoic acid as the directional solvent. The goal is to quantify and identify the sources of inefficiencies in the DSE process. The mass, energy, and exergy balances of the DSE process, which are presented in later sections, are based on the system shown in Figure 2. The thermodynamic properties of seawater mixture are calculated based on the correlations by Sharqawy et al. [20] and modified by Nayar et al. [21]. The thermodynamic properties of the water-solvent binary mixture, on the other hand, were developed and presented hereafter.

## 2. Directional Solvent Extraction (DSE) Desalination Process with Heat Recovery

Alotaibi et al. [8] utilized a heat exchanger network synthesis where a continuous DSE process was designed and optimized for maximum heat recovery. They confirmed that the DSE process with heat recovery using octanoic acid is more energy efficient when compared to a similar process using decanoic acid. Alotaibi et al. [8], however, did not consider an exergy analysis as part of their study. Based on their findings, the present study focuses on performing an exergy analysis of the optimized DSE process flow diagram, as shown in Figure 2. 

Excluding the complexity of the heat exchanger network, the optimized process mainly consists of a mixing tank, two settlement tanks, and six pumps to circulate the seawater, solvent, and hot water. The basic concept behind this process is that water solubility in the directional solvent, octanoic acid in this case, increases with temperature and the directional solvent has a high rate of salt ion rejection. The process starts in the mixing tank where seawater (State 12) is mixed with the directional solvent (State 8). Before entering the mixing tank, both seawater and solvent are heated to a high temperature, which facilitates water to dissolve into the octanoic acid during mixing. The salt concentration in the brine at State 13 is increased as water is absorbed into the solvent. The solvent saturated with pure water becomes separated from concentrated saline water “brine”, in the high-temperature settlement tank, resulting in two separate streams (States 1 and 14). The saturated solvent (State 1) is pumped through heat exchangers to ultimately reduce its temperature to 40 °C at States 4 and 23. The result of the cooling process is an immiscible mixture of saturated directional solvent and pure water. The solvent and pure water, in the low-temperature settlement tank, are separated into two streams. The purified water stream (State 16) is collected and the saturated solvent stream (State 5) is recycled back to the mixing tank. Four heat exchangers are used to preheat the seawater from State 9 to State 21 and the solvent from State 6 to State 7. Three additional heat exchangers are used for the external heating and cooling processes. One heat exchanger is used to cool the solvent-water mixture (State 3) to the lowest process temperature (State 4). The second heat exchanger is utilized to heat the recycled solvent (State 7) to the highest process temperature (State 8). The third heat exchanger is used to heat the seawater from State 21 to the highest process temperature (State 12). A total of six pumps are used to circulate the solvent, seawater, and hot water in the continuous process. 

### 2.1. Thermodynamic Properties of Seawater

The thermodynamic properties of seawater were evaluated using the modified pressure dependence correlations presented by Nayar et al. [21], which are based on the previously developed correlations of Sharqawy et al. [20]. The seawater properties including the specific volume $(v_{sw}$), enthalpy $\left(h_{sw}\right)$, entropy $\left(s_{sw}\right)$ and chemical potential for salt and water $\;\left(\mu_{s},\mu_{w}\right)$, respectively, are given as functions of temperature (T), pressure (P), and salinity (Sa). The seawater flow exergy $\left(\Psi_{sw}\right)$ is then evaluated by the following equation [22]:

(1) $$\Psi_{sw}=\left(h_{sw}-h_{sw}^{*}\right)-T_{0}\left(s_{sw}-s_{sw}^{*}\right)+\;x_{s}\;(\mu_{s}^{*}-\mu_{s}^{o})+\left(1-\;x_{s}\;\right)(\mu_{w}^{*}-\mu_{w}^{o})$$

where $x_{s}$ is the mass fraction of salt in seawater, and subscripts “s”, “w” and “sw” refer to salt, water and seawater, respectively. 

The thermodynamic properties with superscript “*” represent the restricted state, and are evaluated at temperature and pressure of the global dead state ($T_{o},P_{o})$ and at a salinity of the state (*Sa*); while the thermodynamics properties with superscript “^o^” are evaluated at $(T_{o}$, $P_{o})$, and the salinity of the dead state (*Sa_o_*). In this study, the global dead state of seawater is defined as follows:

$$T_{o}=298.15\;K;\;P_{o}=0.101\;\mathrm{MPa};\;Sa_{o}=35\;g/\mathrm{kg}$$

Equation (1) consists of the thermal and chemical exergies. The thermal exergy is achieved when both temperature and pressure changes to those of the environment with no change in concentration. While the chemical exergy is achieved with a change in concentration, but the temperature and pressure are those of the environment.

### 2.2. Thermodynamics Properties of Octanoic Acid-Water Binary Mixtures

**Figure 3:**
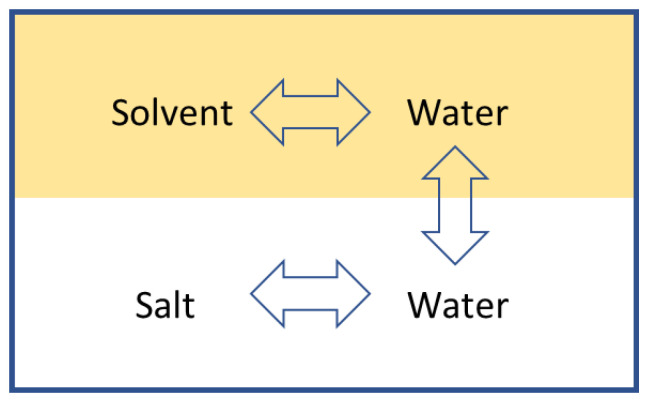
Illustration of the interaction between the seawater and solvent-water binary mixtures in an immiscible binary system.

In the DSE process, there are two binary mixtures. One is the seawater mixture, and the other is the solvent-water mixture. This creates two separate binary mixtures in an immiscible binary system. Figure 3 below represents the whole system where there are interactions between molecules of water and solvent, and molecules of salt and water, but no interaction between the salt and the solvent. The two separate binary systems at equilibrium allow the evaluation of the seawater properties independently from the solvent-water mixture. 

The thermodynamic properties of pure liquid water are evaluated using the correlations by Nayar et al. [21] at zero salt concentration. For the pure liquid solvent, the specific enthalpy, and entropy are evaluated using the following equations:

(2) $$h_{OA}-h_{o}=C_{OA}\cdot \left(T-T_{o}\right)+v_{OA}\cdot \left(P-P_{o}\right)$$

(3) $$s_{OA}-s_{o}=C_{OA}\cdot ln\left(\frac{T}{T_{o}}\right)$$

where *C* is specific heat capacity and v is the specific volume; while subscript “*OA*” refers to the octanoic acid solvent and subscript “*o*” refers to the reference state. The properties of octanoic acid are given in Table 2. In this study, the specific heat and specific volume of octanoic acid are assumed constants. The global dead state, which also used as a reference state, of the solvent–water binary mixture is defined as follows:

$$T_{o}=298.15\;K;\;P_{o}=0.101\;\mathrm{MPa};\;Solu_{o}=10\;g/\mathrm{kg}$$

where $Solu_{o}$ is solubility of water in the directional solvent at the dead state ($T_{o},\;P_{o}).$

The total Gibb’s free energy ($\bar{G}$) of the octanoic acid and water binary mixture is given as follows:

(4) $$\bar{G}=y_{w}\;({\bar{h}}_{w}-T{\bar{s}}_{w})+\left(1-y_{w}\right)({\bar{h}}_{OA}-T\;{\bar{s}}_{OA})+\;\;{\bar{G}}_{mix}+{\bar{G}}^{E}$$

where $y_{w}$ is the mole fraction the of pure water in the solution, ${\bar{G}}_{mix}$ is Gibb’s free energy of mixing and ${\bar{G}}^{E}$ is Gibb’s excess energy. ${\bar{G}}_{mix\;}$ and ${\bar{G}}^{E}\;$ are evaluated using the following equations:

(5) $${\bar{G}}_{mix}=\bar{R}T\left(y_{w}\;lny_{w}+\left(1-y_{w}\right)\ln\left(1-y_{w}\right)\right)$$

(6) $${\bar{G}}^{E}=\bar{R}TAy_{w}\left(1-y_{w}\right)$$

**Figure 4:**
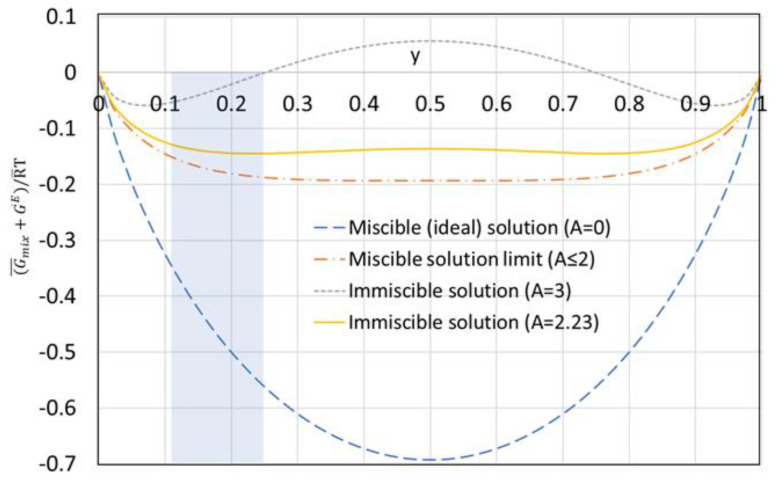
Gibb’s free energy of mixing plus Gibb’s excess energy vs. water mole fraction for different values of the constant *A* in Equation (6).

The above Gibb’s excess energy equation is based on the two-suffix Margules equation [23] which is a simple thermodynamic model for Gibb’s excess free energy. Margules equation was used to predict the behavior of non-ideal immiscible liquid binary mixtures, where *A* is a constant. For ideal miscible solutions *A* = 0; while for non-ideal miscible solutions *A* is greater than zero but less than or equal to two (0 < *A* ≤2). For non-ideal immiscible solutions, *A* is greater than two (*A* > 2). For *A* ≥ 3, the minimum Gibb’s free energy at equilibrium occurs at low water mole fraction outside the range of the solubility of water in octanoic acid as shown in the shaded area in Figure 4. For *A* = 2.23, however, Gibb’s free energy has a flat minimum that covers the solubility of water in octanoic acid for temperatures between 40 °C to 80 °C, corresponding to a water mole fraction between 0.11 and 0.25. In this study, *A* = 2.23 was used to evaluate the thermodynamic properties of the non-ideal octanoic acid–water binary mixtures.

The chemical potential of water ($\mu_{w}$) and octanoic acid ($\mu_{OA}$) in the solution are evaluated using the following equations:

(7) $$\mu_{w}=\left(h_{w}-Ts_{w}\right)+R_{w}T\;ln\left(y_{w}\right)+R_{w}TA{\left(1-y_{w}\right)}^{2}$$

(8) $$\mu_{OA}=\left(h_{OA}-Ts_{OA}\right)+TR_{OA}ln\left(1-y_{w}\;\right)+TR_{OA}Ay_{w}{}^{2}$$

The enthalpy and entropy of the octanoic acid and water binary mixture, $h_{OA,w}\;$ and $s_{OA,w}$, respectively, are calculated using the following equations:

(9) $$h_{OA,w}=x_{w}h_{w}+\left(1-x_{w}\right)h_{OA}$$

(10) $$s_{OA,w}=x_{w}s_{w}+\left(1-x_{w}\right)s_{OA}-RT\left(y_{w}\;lny+\left(1-y_{w}\right)\ln\left(1-y_{w}\right)\right)-RTAy_{w}\left(1-y_{w}\right)$$

where $x_{w}$ is the mass fraction of pure water in the solution.

The exergy of the octanoic acid-water in the solution $\left(\Psi_{OA,w}\right)$ is then evaluated by the following equation:

(11) $$\Psi_{OA,w}=\left(h_{OA,w}-h_{OA,w}^{*}\right)-T_{0}\left(s_{OA,w}-s_{OA,w}^{*}\right)+x_{w}(\mu_{w}^{*}-\mu_{w}^{o})+\left(1-x_{w}\right)(\mu_{OA}^{*}-\mu_{OA}^{o})$$

Similar to Equation (1), the thermodynamic properties with superscript “*” in Equation (11) represent the restricted state, which is evaluated at temperature and pressure of the global dead state ($T_{0},P_{0})$ and at a water solubility of the state (Solu); while the thermodynamics properties with superscript “*o*” are evaluated at $T_{o}$, $P_{o}$, and the water solubility of the dead state (Solu*_o_*).

## 3. Mass, Energy, Entropy, and Exergy Balances

The DSE continuous process given in Figure 2, has two settlement tanks, seven heat exchangers, and six pumps. The mass, energy, and exergy were balanced for each component of the DSE process. The assumptions considered in the mass, energy, and exergy analyses are: (1) the process at steady state; (2) the kinetic and potential energies are neglected; (3) the piping system, pumps, and heat exchangers are well insulated; (4) the mixing and separation are isothermal processes; (5) the purified water (State 16) has no salt concentration; and (6) the concentrated brine (State 14) has no solvent concentration. In this study, the general governing equations for the overall system or sub-systems are given as:

Mass balance

(12) $$\sum_{in}{\dot{m}}_{i}=\underset{out}{\sum }{\dot{m}}_{j}$$

Energy balance

(13) $$\underset{in}{\sum }\left({\dot{m}}_{i}h_{i}\right)+\dot{Q}=\dot{P}+\underset{out}{\sum }\left({\dot{m}}_{j}h_{j}\right)$$

Entropy balance

(14) $$\underset{in}{\sum }{\dot{m}}_{i}s_{i}+\dot{Q}/T_{0}+{\dot{S}}_{gen}=\underset{out}{\sum }{\dot{m}}_{j}s_{j}$$

Exergy balance

(15) $$\underset{in}{\sum }{\dot{m}}_{i}\Psi_{i}+\dot{P}-{\dot{X}}_{d}=\underset{out}{\sum }{\dot{m}}_{j}\Psi_{j}$$

where, $\dot{m},\;h,\;s\;\mathrm{and}\;\Psi$ are the mass flow rate, enthalpy, entropy, exergy at each thermodynamic state, respectively; while $T_{o}$ is surrounding or the dead state temperature, $\dot{Q}$ is the rate of heat transfer, $\dot{P}$ is the rate of power transfer, ${\dot{S}}_{gen}$ is the rate of entropy generation, and ${\dot{X}}_{d}$ is the rate of exergy destruction. Subscripts “*i*” and “*j*” refer to the inlet and outlet streams.

The second-law efficiency, $\eta_{ΙΙ}$, qunatify the performance of irreversible processes. It is defined as the ratio of minimum exergy input required to that of the total actual exergy input [14], which is given as:

(16) $$\eta_{ΙΙ}={\dot{X}}_{min,in}/{\dot{X}}_{\;actual,in}$$

where, ${\dot{X}}_{min,in}$ is calculated for a reversible process as:

(17) $${\dot{X}}_{min,in}=\sum {\dot{m}}_{out}\Psi_{out}-\sum {\dot{m}}_{in}\Psi_{in}$$

**Figure 5:**
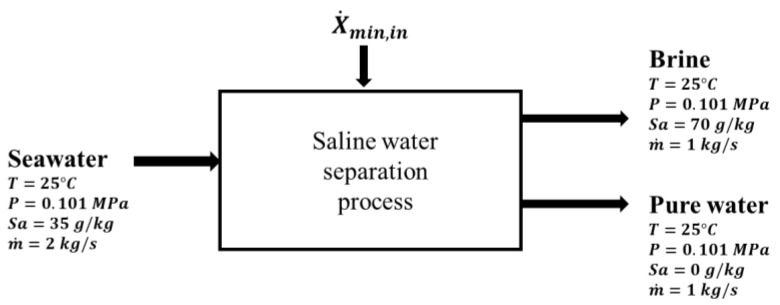
Ideal reversible desalination process at a recovery-ratio of 0.5 [22].

$\Psi_{in}\;\mathrm{and}\;\Psi_{out}$ represent flow exergy of inlet and outlet streams. Figure 5 shows a schematic of a control volume system utilized to evaluate the minimum exergy input using Equation (17) above.

## 4. Results and Discussion

This paper is focused on an exergy analysis of a continuous DSE desalination process using octanoic acid. The thermodynamic properties including the flow of exergy were calculated and listed for each thermodynamic state (e.g., Table A1, Table A2 and Table A3). The mass, energy, and exergy were balanced for different components of the system to quantify and identify the inefficiencies in the process. The exergy destruction of each component of the process is evaluated and presented. A parametric study was performed to evaluate the effect of the critical variables, namely the recovery-ratio, heat exchanger effectiveness, and highest process temperature on the performance of the DSE system. The product water yield measurements for octanoic acid given by Bajepayee [2], shown in Figure 1, were used in the energy and exergy analyses. Bajepayee [2] provided uncertainties for the product water yield measurements in the range of 10% to 36%, which were used to estimate the uncertainties of the calculated values using numerical analysis [24]. 

### 4.1. Effect of Highest Process Temperature and Heat Exchanger Effectiveness

The exergy destructions of the overall system and sub-systems of the process were calculated at different highest process temperature and separate values of heat exchanger effectiveness. Three values of the highest process temperature and three values of the heat exchanger effectiveness were considered in the parametric study.

**Figure 6:**
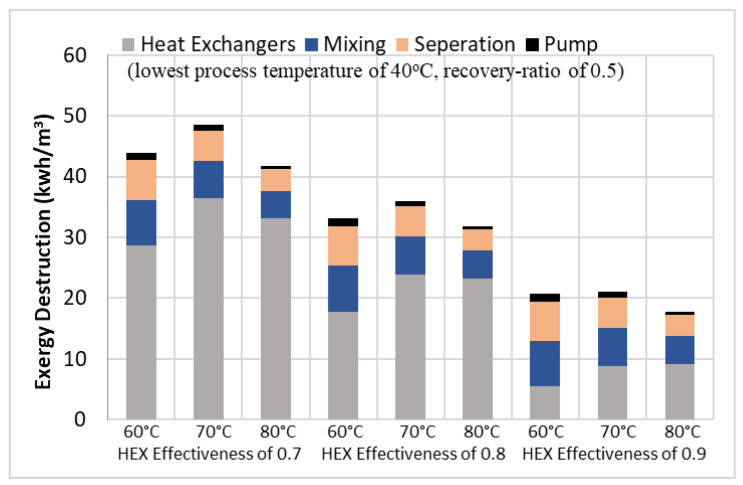
Exergy destruction of various components at different high process temperature and heat exchanger effectiveness.

The results, plotted as bar charts in Figure 6 and presented in Table 3, show the exergy destruction for mixing, separation, and pumps, which have decreasing trends with an increase in the highest process temperature. As the highest temperature increases from 70 °C to 80 °C, the exergy destruction of mixing and separation processes ranges from 7.5 to 4.6 kWh/m^3^ and 6.5 to 3.5 kWh/m^3^, respectively. For the pumps, it ranges from 1.3 to 0.6 kWh/m^3^. The exergy destructions for mixing, separation, and pumps were independent of the heat exchanger effectiveness. Figure 6 also shows that the exergy destruction in the heat exchangers, at the highest process temperature of 60 °C, decreases from 28 kWh/m^3^ to 5 kWh/m^3^ as the heat exchanger effectiveness increases from 0.7 to 0.9. Similar trends were observed for the highest process temperatures of 70 °C and 80 °C, where the exergy destruction decreases from 36 to 9 kWh/m^3^, and from 33 to 9 kWh/m^3^, respectively. For the same heat exchanger effectiveness, there are small variations in the total exergy destruction, within the uncertainty of the calculation, for the different highest process temperatures. The off-trend maximum total exergy destruction at 70 °C can be explained by the relatively low measurement of the product water yield. The uncertainty in the calculated total exergy destruction ranges from 18% to 25%. As the heat exchanger effectiveness increases from 0.7 to 0.9, the percentage contribution of the exergy destruction in the heat exchangers decreases from 79% to 52%; while the percentage contribution of mixing and separation processes increases from 11% to 26% and 8% to 19%, respectively. 

**Figure 7:**
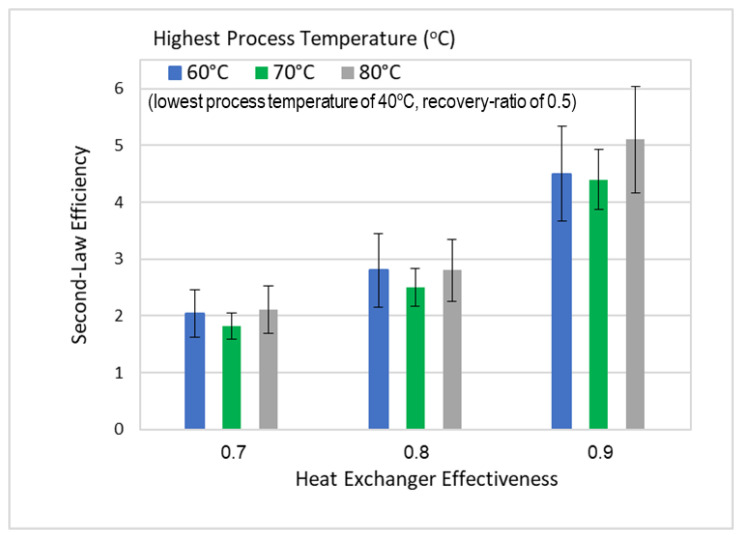
Second-law efficiency at different high process temperature and heat exchanger effectiveness.

Using Equations (16) and (17), the second-law efficiency for the DSE process was evaluated and presented at different heat exchanger effectiveness and different highest process temperatures, but at the same lowest and highest process temperatures of 40 °C and 80 °C, as shown in Figure 7. It is noticed that as the heat exchanger effectiveness increases the second-law efficiency increases from about 2% to 5%, with uncertainty ranges from ± 0.5% to ± 1%. The off-trend minimum value of the second-law efficiency at 70 °C can also be explained by the relatively low measurement of the product water yield.

### 4.2. Effect of Recovery-Ratio and Heat Exchanger Effectiveness

**Figure 8:**
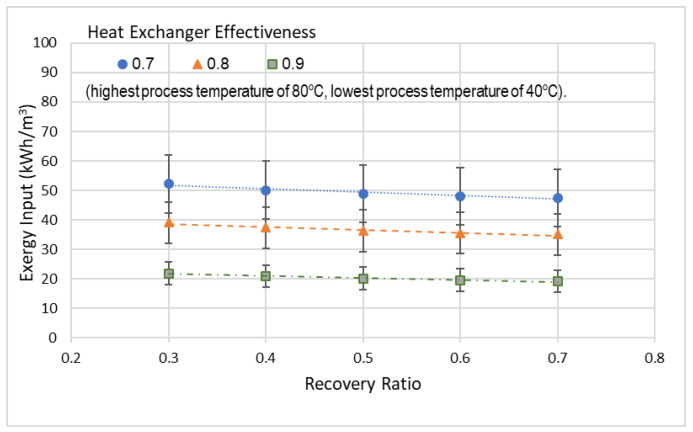
Total exergy input at different recovery-ratio and heat exchanger effectiveness.

Figure 8 displays the total exergy input vs. recovery-ratio, which is plotted at separate values of heat exchanger effectiveness, but at the same lowest and highest process temperatures of 40 °C and 80 °C. Figure 8 shows that the total exergy input at different values of recovery-ratio has a slightly decreasing trend with maximum and minimum being at 0.3 and 0.7, respectively. Also, there is a reduction of about 5 kWh/m^3^ of exergy input, when the recovery-ratio increases from 0.3 to 0.7 for all heat exchanger effectiveness. The decrease in the total exergy input with an increase in recovery-ratio is due to the decrease in the mass flow of seawater, which leads to lower exergy consumption. By observing the graph pattern, it is revealed that at 0.3 recovery-ratio the increase in the heat exchanger effectiveness from 0.7 to 0.9 will lead to a decrease in the total exergy input from 52 to 22 kWh/m^3^. The uncertainty in the total exergy input presented in Figure 8 is about ± 18%.

### 4.3. Performance Comparison with Traditional Desalination Processes

**Figure 9:**
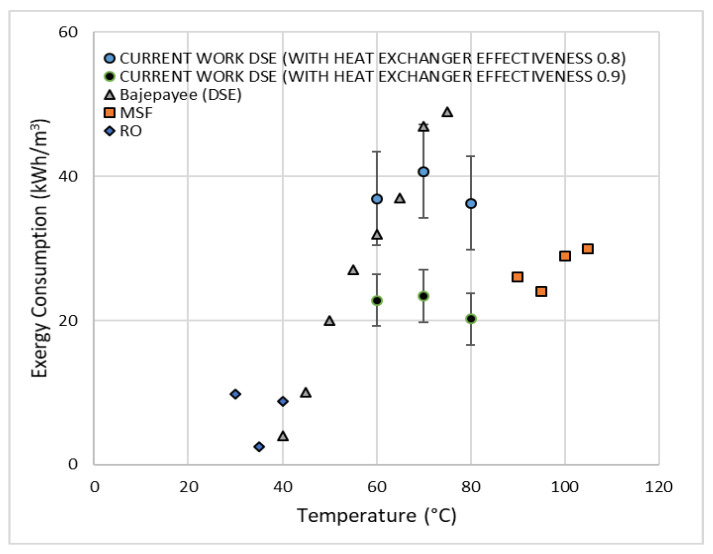
Exergy consumption of different desalination processes at different temperatures.

Figure 9 shows the exergy consumption of different desalination processes at different temperatures. The exergy data for RO and MSF is taken from the literature survey provided in reference [2]. The exergy consumption of RO is in the range of 2.5 to 10 kWh/m^3^ for the temperatures from 30 °C to 40 °C. While, for MSF, it has a maximum value of 33 kWh/m^3^ at 110 °C and a minimum of 24 kWh/m^3^ at 95 °C. The exergy consumption of DSE process using octanoic acid evaluated in reference [2] was in the range of 35 kWh/m^3^ at 60 °C to 49 kWh/m^3^ at approximately 80 °C. For the highest process temperature of 60 °C, 70 °C, and 80 °C considered in the current study, the exergy consumption ranges from 36 to 41 kWh/m^3^, for heat effectiveness of 0.8 and a recovery-ratio of 0.5. The results of Bajeypee [2] and the current work are comparable and in the same range within the uncertainty of the exergy calculation. When compared to the traditional desalination processes, DSE exergy consumption is much higher than RO but is comparable to MSF when the heat exchanger effectiveness is at 0.9. 

## 5. Conclusions

An exergy analysis was performed on a DSE system, using process modeling, to predict its performance. Four primary sources of exergy destruction were identified and evaluated, namely: mixing, separation, heat exchangers, and pumps. The results show that the heat exchangers have the highest exergy destruction. The results also reveal that the total exergy input decreases from around 50 kWh/m^3^ to less than 20 kWh/m^3^ with an increase in heat exchanger effectiveness from 0.7 to 0.9. The total exergy input, however, has a slightly decreasing trend of less than 5 kWh/m^3^ as the recovery-ratio increases from 0.3 to 0.7. The exergy consumption of the DSE process with high heat recovery of 90% is comparable to those of MSF and much higher than RO. Octanoic acid, the directional solvent used in this study, has lower product water yield that causes higher exergy consumption. Therefore, to take advantage of the relatively low-temperature operation of the DSE desalination process, more effective directional solvents with higher yield are required to make it more competitive.

## Figures and Tables

**Table 1:** Electrical energy consumption of mixing and separation [8].

	Electrical Energy Consumption (kWh/m^3^)	
*T* (°C)	Mixing	Separation
60	0.222	3.26
70	0.093	2.53
80	0.026	1.74

**Table 2:** Properties of octanoic acid [2].

Directional Solvent	Specific Volume (*v*) (m^3^/kg)	Specific Heat (*C_p_*)(kJ/kg∙K)	Melting Point (°C)
Octanoic Acid	0.001098	2.2	25

**Table 3:** Exergy destruction of various components at different high process temperature and heat exchanger effectiveness, (low process temperature of 40 °C, recovery-ratio of 0.5). These values are also shown as bar charts in Figure 6 above.

Heat Exchanger Effectiveness	0.7			0.8			0.9		
High Process Temperature	60 °C	70 °C	80 °C	60 °C	70 °C	80 °C	60 °C	70 °C	80 °C
Mixing	7.5	6.2	4.6	7.5	6.2	4.6	7.5	6.2	4.6
Separation	6.5	5	3.5	6.5	5	3.5	6.5	5	3.5
Heat Exchangers	28.7	36.4	33.1	17.8	23.9	23.2	5.5	8.9	9.2
Pump	1.3	0.9	0.6	1.3	0.9	0.6	1.2	0.9	0.6
Total Exergy Destruction	44.1	48.6	42	33.1	36.1	31.9	20.1	21.2	18

## Nomenclature

**Abbreviations**	
DSE	Directional Solvent Extraction
HEX	Heat Exchanger
MED	Multi-Effect Distillation
MSF	Multi-Stage Flashing
RO	Reverse Osmosis
**Symbols**	
$\dot{Q}$	Heat transfer (kW)
$\dot{m}$	Mass flow rate (kg/s)
$\bar{h}$	Enthalpy (kJ/kmole)
$\bar{R}$	Universal gas constant (kJ/kmol-K)
R	Gas constant (kJ/kg-K)
$\bar{s}$	Entropy (kJ/kmole-K)
s	Entropy (kJ/kg-K)
$\bar{G}$	Gibbs free energy (kJ/kmole)
*T*	Temperature (°C)
*x*	Mass fraction
*y*	Mole fraction
*Sa*	Salinity (g/kg)
$\dot{P}$	Power (kW)
**Subscripts**	
*d*	Destruction
*gen*	Generation
*in*	Inlet
*mix*	Mixing
*o*	Dead state
*OA*	Octanoic acid
*out*	Outlet
*s*	Solvent
*sw*	Seawater
*w*	Water
**Superscripts**	
*	Restricted state
^o^	Dead state
E	Excess
**Greek Symbols**	
Ψ	Flow exergy (kJ/kg)
*µ*	Chemical potential (kJ/kg)
$\eta_{ΙΙ}$	Second-law efficiency

## Appendix A

**Table A1:** Enthalpy, entropy, and exergy at each state of DSE process (highest process temperature = 80 °C).

State	*T* (°C)	*P* (kPa)	$\dot{m}$ (kg/s)	h (kJ/kg)	s (kJ/kg·K)	Ψ (kJ/kg)	Description *
1	80	101	38.27	129.77	0.4103	8.798	OA/W
2	80	151	37.22	129.84	0.4103	8.856	OA/W
3	47	126	37.22	54.48	0.1852	0.601	OA/W
4	40	101	37.22	38.52	0.1343	−0.164	OA/W + W
5	40	101	37.27	35.06	0.1225	−0.279	OA/W
6	40	151	37.27	35.13	0.1226	−0.223	OA/W
7	74	126	37.27	110.39	0.3509	6.965	OA/W
8	80	101	37.27	124.26	0.3906	8.997	OA/W
9	25	101	2.00	99.77	0.3498	0.000	SW
10	25	151	1.40	99.83	0.3499	0.049	SW
11	59	141	1.40	234.18	0.7770	7.051	SW
12	80	101	2.00	320.50	1.0294	18.140	SW
13	80	101	39.27	134.27	0.4246	9.047	OA/W + CB
14	80	121	1.00	306.72	0.9720	18.600	CB
15	31	101	1.00	118.23	0.3978	1.322	CB
16	40	121	1.00	167.62	0.5724	4.114	FW
17	35	101	1.00	146.73	0.5052	3.274	FW
18	25	151	0.60	99.83	0.3499	0.049	SW
19	34	141	0.60	134.83	0.4657	0.512	SW
20	74	126	0.60	295.44	0.9577	14.460	SW
21	63	126	2.00	252.46	0.8318	9.000	SW
22	80	151	1.05	129.84	0.4103	8.856	OC/W
23	40	101	1.05	38.52	0.1343	−0.164	OC/W + W
24	25	101	12.42	99.77	0.3498	0.000	SW
25	25	151	12.42	99.83	0.3499	0.049	SW
26	37	101	12.42	147.66	0.5075	0.901	SW
27	90	101	19.70	376.91	1.1926	28.480	HW
28	90	151	19.70	376.98	1.1927	28.540	HW
29	84	101	19.70	350.74	1.1199	24.000	HW
30	90	101	1.92	376.91	1.1926	28.480	HW
31	90	151	1.92	376.98	1.1927	28.540	HW
32	73	101	1.92	305.97	0.9925	17.220	HW

* OA = Octanoic Acid, W = Water, SW = Seawater (Sa = 35 g/kg), CB = Concentrated Brine (Sa = 70 g/kg), FW = Fresh water, HW = Hot Water.

**Table A2:** Enthalpy, entropy, and exergy at each state of DSE process (highest process temperature = 70 °C).

State	*T* (°C)	*P* (kPa)	$\dot{m}$ (kg/s)	h (kJ/kg)	s (kJ/kg·K)	Ψ (kJ/kg)	Description *
1	70	101	60.74	105.108	0.3383	5.530	OA/W
2	70	151	59.81	105.184	0.3384	5.587	OA/W
3	45	126	59.81	49.496	0.1692	0.341	OA/W
4	40	101	59.81	37.238	0.1299	−0.206	OA/W + Water
5	40	101	59.74	35.055	0.1225	−0.279	OA/W
6	40	151	59.74	35.131	0.1226	−0.223	OA/W
7	65	126	59.74	90.888	0.294	4.438	OA/W
8	70	101	59.74	101.962	0.3266	5.795	OA/W
9	25	101	2.00	99.765	0.3498	0.000	SW
10	25	151	1.48	99.833	0.3499	0.049	SW
11	51	141	1.48	204.459	0.6864	4.343	SW
12	70	101	2.00	280.247	0.9137	12.370	SW
13	70	101	61.74	107.746	0.3468	5.651	OA/W + Brine
14	70	121	1.00	268.039	0.8609	13.050	CB
15	30	101	1.00	113.423	0.3819	1.239	CB
16	40	121	1.00	167.624	0.5724	4.114	FW
17	35	101	1.00	146.730	0.5052	3.274	FW
18	25	151	0.52	99.833	0.3499	0.049	SW
19	35	141	0.52	139.845	0.482	0.663	SW
20	65	126	0.52	260.239	0.8549	9.900	SW
21	55	126	2.00	219.023	0.7311	5.581	SW
22	70	151	0.93	105.184	0.3384	5.587	OC/W
23	40	101	0.93	37.238	0.1299	−0.206	OC/W + Water
24	25	101	17.66	99.765	0.3498	0.000	SW
25	25	151	17.66	99.833	0.3499	0.049	SW
26	35	101	17.66	141.345	0.487	0.674	SW
27	80	101	31.60	334.931	1.075	21.470	HW
28	80	151	31.60	335.002	1.075	21.530	HW
29	75	101	31.60	314.070	1.016	18.360	HW
30	80	101	1.92	334.931	1.075	21.470	HW
31	80	151	1.92	335.002	1.075	21.530	HW
32	65	101	1.92	271.158	0.890	12.760	HW

* OA = Octanoic Acid, W = Water, SW = Seawater (Sa = 35 g/kg), CB = Concentrated Brine (Sa = 70 g/kg), FW = Fresh water, HW = Hot Water.

**Table A3:** Enthalpy, entropy, and exergy at each state of DSE process (highest process temperature = 60 °C).

State	*T* (°C)	*P* (kPa)	$\dot{m}$ (kg/s)	h (kJ/kg)	s (kJ/kg·K)	Ψ (kJ/kg)	Description *
1	60	101	85.63	81.662	0.2684	2.895	OA/W
2	60	151	84.83	81.738	0.2685	2.952	OA/W
3	44	126	84.83	44.749	0.1540	0.107	OA/W
4	40	101	84.83	36.603	0.1278	−0.227	OA/W + W
5	40	101	84.63	35.055	0.1225	−0.279	OA/W
6	40	151	84.63	35.131	0.1226	−0.223	OA/W
7	57	126	84.63	72.208	0.2380	2.434	OA/W
8	60	101	84.63	79.659	0.2606	3.159	OA/W
9	25	101	2.00	99.765	0.3498	0.000	SW
10	25	151	1.55	99.833	0.3499	0.049	SW
11	45	141	1.55	178.576	0.6058	2.493	SW
12	60	101	2.00	240.017	0.7947	7.618	SW
13	60	101	86.63	83.366	0.2739	2.959	OA/W + CB
14	60	131	1.00	229.306	0.7464	8.465	CB
15	28	121	1.00	107.194	0.3613	1.162	CB
16	40	101	1.00	167.624	0.5724	4.114	FW
17	35	101	1.00	146.730	0.5052	3.274	FW
18	25	151	0.45	99.833	0.3499	0.049	SW
19	37	141	0.45	146.343	0.5031	0.889	SW
20	57	126	0.45	226.558	0.7540	6.286	SW
21	47	126	2.00	189.354	0.6396	3.191	SW
22	60	151	0.80	81.738	0.2685	2.952	OA/W
23	40	101	0.80	36.603	0.1278	−0.227	OA/W + W
24	25	101	20.15	99.765	0.3498	0.000	SW
25	25	151	20.15	99.833	0.3499	0.049	SW
26	34	101	20.15	134.134	0.4636	0.455	SW
27	70	101	44.70	293.036	0.9549	15.480	HW
28	70	151	44.70	293.107	0.9550	15.530	HW
29	67	101	44.70	279.003	0.9138	13.700	HW
30	70	101	1.92	293.036	0.9549	15.480	HW
31	70	151	1.92	293.107	0.9550	15.530	HW
32	57	101	1.92	240.258	0.7982	9.436	HW

* OA = Octanoic Acid, W = Water, SW = Seawater (Sa = 35 g/kg), CB = Concentrated Brine (Sa = 70 g/kg), FW = Fresh water, HW = Hot Water.

## References

[1] Davidson R.R., Smith W.H., Hood D.W. (1960). Structure and Amine-water Solubility in Desalination by Solvent Extraction. J. Chem. Eng. Data.

[2] Bajpayee A. (2012). Directional Solvent Extraction Desalination. Ph.D. Thesis.

[3] Bajpayee A., Luo T., Muto A., Chen G. (2011). Very low-temperature Membrane-free Desalination by Directional Solvent Extraction. Energy Environ. Sci..

[4] Luo T., Bajpayee A., Chen G. (2011). Directional Solvent for Membrane-free Water Desalination—A Molecular Study. J. Appl. Phys..

[5] Rish D., Luo S., Kurtz B., Luo T. (2014). Exceptional Ion Rejection Ability of Directional Solvent for Non-Membrane Desalination. Appl. Phys. Lett..

[6] Sanap B.D., Kadam D.K., Narayan M., Kasthurirangan S., Nemade R.P., Dalvi V.H. (2015). Analysis of Saline Water Desalination by Directed Solvent Extraction using octanoic acid. Desalination.

[7] Luo S., Pang Y., Luo T. (2018). A Continuous Directional Solvent Extraction Desalination Process Realized with the Aid of Electro Coalescence. J. Chem. Eng. Process Technol..

[8] Alotaibi S., Ibrahim O.M., Luo S., Luo T. (2017). Modeling of a Continuous Water Desalination Process Using Directional Solvent Extraction. Desalination.

[9] Kahraman N., Cengel Y.A., Wood B., Cerci Y. (2005). Exergy Analysis of a Combined RO, NF, and EDR Desalination Plant. Desalination.

[10] Hou S., Zeng D., Ye S., Zhang H. (2007). Exergy Analysis of the Solar Multi-effect Humidification-dehumidification Desalination Process. Desalination.

[11] Mokhtari H., Sepahvand M., Fasihfar A. (2016). Thermo-economic, and Exergy Analysis in Using Hybrid Systems (GT+MED+RO) for Desalination of Brackish Water in Persian Gulf. Desalination.

[12] Elsayed M.L., Mesalhy O., Mohammed R.H., Chow L.C. (2019). Performance Modeling of MED-MVC Systems: Exergy-economic analysis. Energy.

[13] Cerci Y. (2002). Exergy Analysis of a Reverse Osmosis Desalination Plant in California. Desalination.

[14] Kahraman N., Cengel Y.A. (2005). Exergy Analysis of a MSF Distillation Plant. Energy Convers. Manag..

[15] Nafey A.S., Fath H.E.S., Mabrouk A.A. (2008). Thermo-economic Design of a Multi-effect Evaporation Mechanical Vapor Compression (MEE-MVC) Desalination Process. Desalination.

[16] Banat F., Jwaied N. (2008). Exergy Analysis of Desalination by Solar-powered Membrane Distillation Units. Desalination.

[17] Al Ghamdi A., Mustafa I. (2016). Exergy Analysis of a MSF Desalination Plant in Yanbu, Saudi Arabian. Desalination.

[18] Mistry K. (2008). Second-Law Analysis, and Optimization of Humidification-Dehumidification Desalination Cycles. Ph.D. Thesis.

[19] Chen Q., Ja M.K., Li Y., Chua K.J. (2017). On the Second-law Analysis of a Multi-Stage Spray-assisted Low-temperature Desalination System. Energy Convers. Manag..

[20] Sharqawy M.H., Lienhard J.H., Zubair S.M. (2010). Thermophysical Properties of Seawater: A Review of Existing Correlations and Data. Desalin. Water Treat..

[21] Nayar K.G., Sharqawy M.H., Banchik L.D., Lienhard J.H. (2016). Thermophysical Properties of Seawater: A Review and New Correlations that Include Pressure Dependence. Desalination.

[22] Sharqawy M.H., Zubair S.M., Lienhard J.H. (2011). Second-Law Analysis of Reverse Osmosis Desalination Plants: An Alternative Design Using Pressure Retarded Osmosis. Energy.

[23] Winnick J. (1997). Chemical Engineering Thermodynamics.

[24] Klein S.A. Engineering Equation Solver, Academic Professional, Version 8. http://www.fchart.com/.

